# *N*-(2,3,4-Trifluoro­phen­yl)pyrrolidine-1-carboxamide

**DOI:** 10.1107/S1600536811051385

**Published:** 2011-12-03

**Authors:** Shuchen Pei, Jie Li, Boyi Qu, Li Hai, Yong Wu

**Affiliations:** a Key Laboratory of Drug Targeting of the Education Ministry, West China School of Pharmacy, Sichuan University, Chengdu 610041, People’s Republic of China

## Abstract

In the title compound, C_11_H_11_F_3_N_2_O, a urea derivative, the best plane through the pyrrole ring makes a dihedral angle of 9.69 (13)° with the benzene ring. The amino H atom is shielded, so that it is not involved in any hydrogen-bonding inter­actions.

## Related literature

For background to this class of compounds, see Zheng *et al.* (2010 ▶).
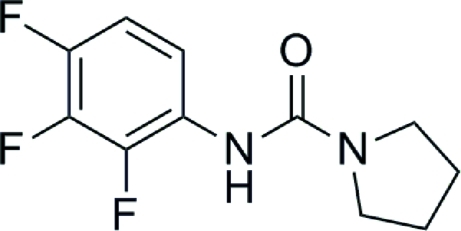


## Experimental

### 

#### Crystal data

C_11_H_11_F_3_N_2_O
*M* *_r_* = 244.22
Monoclinic, 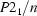

*a* = 6.0708 (4) Å
*b* = 24.2124 (15) Å
*c* = 7.4232 (6) Å
β = 100.508 (7)°
*V* = 1072.83 (13) Å^3^
*Z* = 4
Mo *K*α radiation
μ = 0.13 mm^−1^
*T* = 293 K
0.35 × 0.35 × 0.25 mm

#### Data collection

Oxford Diffraction Xcalibur Eos diffractometer
Absorption correction: multi-scan (*CrysAlis PRO*; Oxford Diffraction, 2007 ▶) *T*_min_ = 0.964, *T*_max_ = 1.000
4465 measured reflections
2190 independent reflections
1374 reflections with *I* > 2σ(*I*)
*R*_int_ = 0.018

#### Refinement

*R*[*F* ^2^ > 2σ(*F* ^2^)] = 0.054
*wR*(*F* ^2^) = 0.151
*S* = 1.04
2190 reflections
158 parameters
H atoms treated by a mixture of independent and constrained refinement
Δρ_max_ = 0.24 e Å^−3^
Δρ_min_ = −0.22 e Å^−3^

### 

Data collection: *CrysAlis PRO* (Oxford Diffraction, 2007 ▶); cell refinement: *CrysAlis PRO*; data reduction: *CrysAlis PRO*; program(s) used to solve structure: *SHELXS97* (Sheldrick, 2008 ▶); program(s) used to refine structure: *SHELXL97* (Sheldrick, 2008 ▶); molecular graphics: *OLEX2* (Dolomanov *et al.*, 2009 ▶); software used to prepare material for publication: *OLEX2*.

## Supplementary Material

Crystal structure: contains datablock(s) global, I. DOI: 10.1107/S1600536811051385/bt5716sup1.cif

Structure factors: contains datablock(s) I. DOI: 10.1107/S1600536811051385/bt5716Isup2.hkl

Supplementary material file. DOI: 10.1107/S1600536811051385/bt5716Isup3.cml

Additional supplementary materials:  crystallographic information; 3D view; checkCIF report

## Figures and Tables

**Fig. 1.**
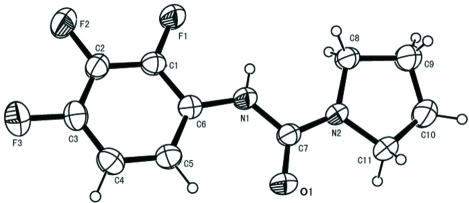
The molecular structure of the title compound, with displacement ellipsoids drawn at the 50% probability level.

**Fig. 2.**
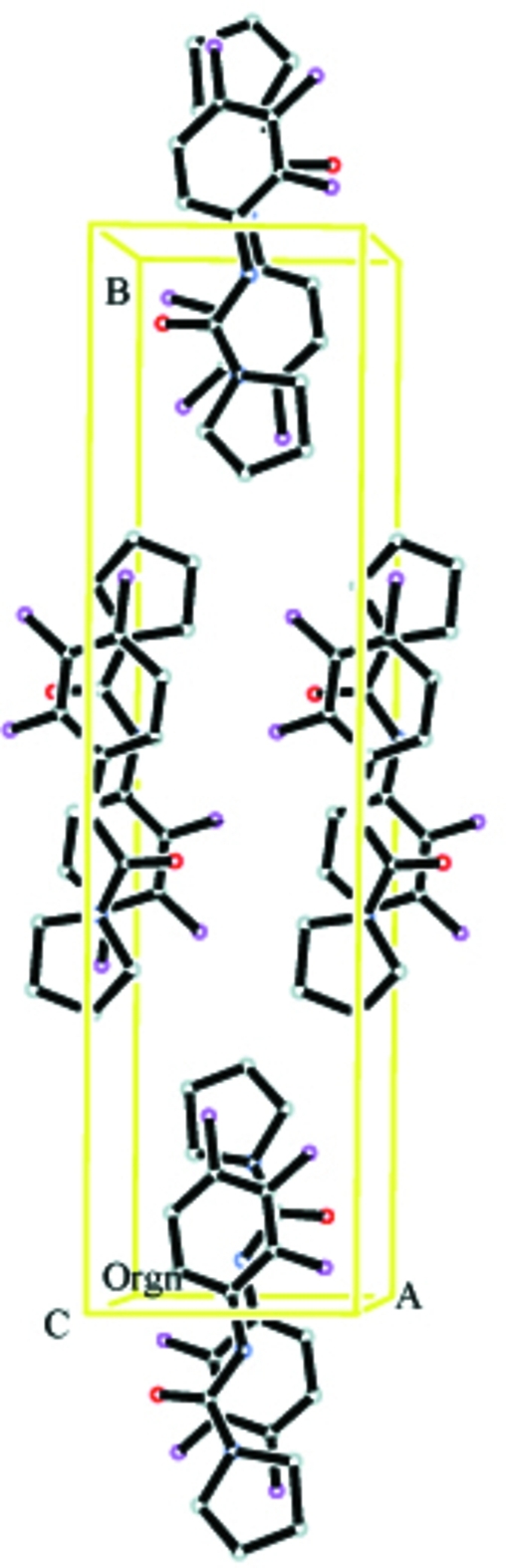
A packing diagram for the title compound.

## supplementary crystallographic information

### Comment

The compound *N*-(2,3,4-trifluorophenyl)pyrrolidine-1-carboxamide is one of urea derivatives. It has been established that urea derivatives have got a significant placein modern medicinal chemistry. Urea derivatives have been reported in the literature as anticancer agent, anticonvulsant, CXCR3 antagonist,antibacterial and so on. Our interests in synthesizing urea derivatives prompted us to develop an efficient methodology for synthesizing *N*-(2,3,4-trifluorophenyl)pyrrolidine-1-carboxamide. In our synthetic work, we obtained the title compound, and its crystal structure is reported here. The three fluorine atoms of the attached benzene ring are close to being coplanar with the ring, whereas the pyrrole ring is not coplanar with the benzene ring.

### Experimental

The title compound was obtained as a derivative of urea. To a solution of triphosgene (350 mg, 1.19 mmol) and triethylamine (680 mg, 6.80 mmol) in anhydrous acetonitrile (5 ml) at ice bath, the solution of 2,3,4-trifluoroaniline (500 mg, 3.40 mmol) and triethylamine (680 mg, 6.80 mmol) in anhydrous acetonitrile (5 ml) were added dropwise.The mixture was stirred for 1 h. And then the solition of tetrahydropyrrole (240 mg,3.40 mmol) and triethylamine (680 mg, 6.80 mmol) in anhydrous acetonitrile (5 ml) were added dropwise. The reaction mixture was then removed from the cooling bath and stirred at room temperature overnight. On completion of the reaction, the mixture was poured into water. The aqueous layer was extracted with ethyl acetate and the organic layer was separated. The organic layers were washed with brine and dried over sodium sulfate, filtered, and concentrated *in vacuo*. The purification of the residue by silica gel column chromatography eluting with EtOAc-petroleum ether (1:10) yielded the white solid 660 mg (yield 86.7%) of *N*-(2,3,4-trifluorophenyl)pyrrolidine-1-carboxamide. Colorless crystals suitable for X-ray analysis were obtained by slow evaporation in ethyl acetate at room temperature.

### Refinement

H atoms bonded to C were positioned geometrically (C—H = 0.93–0.97 Å) and refined using a riding model, with *U*_iso_(H) = 1.2*U*_eq_(C). The amino H atom was freely refined.

### Crystal data

**Table:**

C_11_H_11_F_3_N_2_O	*F*(000) = 504
*M**_r_* = 244.22	*D*_x_ = 1.512 Mg m^−^^3^
Monoclinic, *P*2_1_/*n*	Mo *K*α radiation, λ = 0.7107 Å
*a* = 6.0708 (4) Å	Cell parameters from 1445 reflections
*b* = 24.2124 (15) Å	θ = 2.9–28.9°
*c* = 7.4232 (6) Å	µ = 0.13 mm^−^^1^
β = 100.508 (7)°	*T* = 293 K
*V* = 1072.83 (13) Å^3^	Block, colorless
*Z* = 4	0.35 × 0.35 × 0.25 mm

### Data collection

**Table:**

Oxford Diffraction Xcalibur Eos diffractometer	2190 independent reflections
Radiation source: Enhance (Mo) X-ray Source	1374 reflections with *I* > 2σ(*I*)
graphite	*R*_int_ = 0.018
Detector resolution: 16.0874 pixels mm^-1^	θ_max_ = 26.4°, θ_min_ = 2.9°
ω scans	*h* = −7→6
Absorption correction: multi-scan (*CrysAlis PRO*; Oxford Diffraction, 2007)	*k* = −30→27
*T*_min_ = 0.964, *T*_max_ = 1.000	*l* = −8→9
4465 measured reflections	

### Refinement

**Table:**

Refinement on *F*^2^	Primary atom site location: structure-invariant direct methods
Least-squares matrix: full	Secondary atom site location: difference Fourier map
*R*[*F*^2^ > 2σ(*F*^2^)] = 0.054	Hydrogen site location: inferred from neighbouring sites
*wR*(*F*^2^) = 0.151	H atoms treated by a mixture of independent and constrained refinement
*S* = 1.04	*w* = 1/[σ^2^(*F*_o_^2^) + (0.055*P*)^2^ + 0.3084*P*] where *P* = (*F*_o_^2^ + 2*F*_c_^2^)/3
2190 reflections	(Δ/σ)_max_ < 0.001
158 parameters	Δρ_max_ = 0.24 e Å^−^^3^
0 restraints	Δρ_min_ = −0.22 e Å^−^^3^

### Special details

**Table:**

Geometry. All e.s.d.'s (except the e.s.d. in the dihedral angle between two l.s. planes) are estimated using the full covariance matrix. The cell e.s.d.'s are taken into account individually in the estimation of e.s.d.'s in distances, angles and torsion angles; correlations between e.s.d.'s in cell parameters are only used when they are defined by crystal symmetry. An approximate (isotropic) treatment of cell e.s.d.'s is used for estimating e.s.d.'s involving l.s. planes.
Refinement. Refinement of *F*^2^ against ALL reflections. The weighted *R*-factor *wR* and goodness of fit *S* are based on *F*^2^, conventional *R*-factors *R* are based on *F*, with *F* set to zero for negative *F*^2^. The threshold expression of *F*^2^ > σ(*F*^2^) is used only for calculating *R*-factors(gt) *etc*. and is not relevant to the choice of reflections for refinement. *R*-factors based on *F*^2^ are statistically about twice as large as those based on *F*, and *R*- factors based on ALL data will be even larger.

### Fractional atomic coordinates and isotropic or equivalent isotropic displacement parameters (Å^2^)

**Table:**

	*x*	*y*	*z*	*U*_iso_*/*U*_eq_	
F1	0.1373 (2)	−0.04074 (6)	0.1339 (2)	0.0855 (6)	
F2	0.2120 (3)	−0.14684 (7)	0.2355 (3)	0.0987 (7)	
F3	0.6185 (3)	−0.17950 (6)	0.4120 (2)	0.0908 (6)	
O1	0.7849 (3)	0.08144 (8)	0.2954 (3)	0.0827 (6)	
N1	0.4504 (4)	0.03783 (9)	0.2033 (3)	0.0586 (6)	
H1	0.325 (4)	0.0407 (10)	0.159 (4)	0.061 (9)*	
N2	0.4818 (3)	0.13161 (8)	0.1745 (3)	0.0577 (6)	
C1	0.3428 (4)	−0.05587 (11)	0.2237 (3)	0.0577 (6)	
C2	0.3786 (5)	−0.10989 (11)	0.2741 (4)	0.0626 (7)	
C3	0.5855 (5)	−0.12546 (11)	0.3645 (4)	0.0649 (7)	
C4	0.7542 (5)	−0.08815 (11)	0.4034 (4)	0.0676 (7)	
H4	0.8950	−0.0992	0.4640	0.081*	
C5	0.7159 (4)	−0.03335 (11)	0.3522 (4)	0.0645 (7)	
H5	0.8315	−0.0078	0.3799	0.077*	
C6	0.5075 (4)	−0.01615 (10)	0.2603 (3)	0.0521 (6)	
C7	0.5845 (4)	0.08395 (10)	0.2289 (3)	0.0558 (6)	
C8	0.2441 (4)	0.13970 (10)	0.1006 (4)	0.0637 (7)	
H8A	0.1513	0.1238	0.1807	0.076*	
H8B	0.2032	0.1234	−0.0203	0.076*	
C9	0.2219 (5)	0.20173 (12)	0.0924 (5)	0.0955 (11)	
H9A	0.1181	0.2128	−0.0170	0.115*	
H9B	0.1664	0.2153	0.1987	0.115*	
C10	0.4431 (5)	0.22379 (13)	0.0893 (5)	0.0987 (11)	
H10A	0.4614	0.2594	0.1501	0.118*	
H10B	0.4652	0.2284	−0.0359	0.118*	
C11	0.6072 (5)	0.18321 (11)	0.1877 (4)	0.0744 (8)	
H11A	0.7371	0.1800	0.1293	0.089*	
H11B	0.6562	0.1939	0.3146	0.089*	

### Atomic displacement parameters (Å^2^)

**Table:**

	*U*^11^	*U*^22^	*U*^33^	*U*^12^	*U*^13^	*U*^23^
F1	0.0581 (10)	0.0683 (10)	0.1208 (14)	−0.0094 (8)	−0.0083 (9)	0.0058 (9)
F2	0.0865 (13)	0.0638 (10)	0.1378 (17)	−0.0188 (9)	−0.0004 (11)	0.0062 (10)
F3	0.1087 (15)	0.0639 (11)	0.0979 (14)	0.0123 (9)	0.0135 (10)	0.0105 (9)
O1	0.0462 (11)	0.0746 (13)	0.1207 (18)	−0.0060 (9)	−0.0020 (10)	0.0012 (11)
N1	0.0477 (13)	0.0583 (14)	0.0662 (15)	−0.0060 (11)	0.0010 (11)	−0.0014 (10)
N2	0.0468 (12)	0.0521 (12)	0.0723 (14)	−0.0087 (10)	0.0060 (10)	−0.0041 (10)
C1	0.0524 (15)	0.0623 (16)	0.0570 (15)	−0.0028 (13)	0.0064 (11)	−0.0034 (12)
C2	0.0659 (17)	0.0541 (16)	0.0686 (17)	−0.0082 (14)	0.0147 (13)	−0.0042 (13)
C3	0.082 (2)	0.0562 (16)	0.0581 (16)	0.0061 (15)	0.0158 (14)	−0.0003 (12)
C4	0.0633 (17)	0.0739 (18)	0.0628 (17)	0.0100 (15)	0.0039 (13)	0.0012 (14)
C5	0.0563 (16)	0.0664 (17)	0.0675 (17)	−0.0033 (14)	0.0029 (12)	−0.0041 (13)
C6	0.0538 (15)	0.0576 (15)	0.0451 (14)	−0.0014 (12)	0.0097 (11)	−0.0049 (11)
C7	0.0506 (15)	0.0584 (15)	0.0588 (15)	−0.0065 (13)	0.0112 (11)	−0.0061 (12)
C8	0.0516 (15)	0.0632 (16)	0.0736 (18)	−0.0067 (13)	0.0045 (12)	0.0055 (13)
C9	0.069 (2)	0.069 (2)	0.144 (3)	−0.0005 (17)	0.007 (2)	0.0207 (19)
C10	0.082 (2)	0.0619 (19)	0.146 (3)	−0.0105 (18)	0.003 (2)	0.0102 (19)
C11	0.0585 (17)	0.0621 (17)	0.101 (2)	−0.0144 (14)	0.0086 (15)	−0.0058 (15)

### Geometric parameters (Å, °)

**Table:**

F1—C1	1.353 (3)	C4—C5	1.388 (4)
F2—C2	1.341 (3)	C5—H5	0.9300
F3—C3	1.360 (3)	C5—C6	1.387 (3)
O1—C7	1.228 (3)	C8—H8A	0.9700
N1—H1	0.77 (3)	C8—H8B	0.9700
N1—C6	1.398 (3)	C8—C9	1.508 (4)
N1—C7	1.375 (3)	C9—H9A	0.9700
N2—C7	1.338 (3)	C9—H9B	0.9700
N2—C8	1.461 (3)	C9—C10	1.449 (4)
N2—C11	1.457 (3)	C10—H10A	0.9700
C1—C2	1.367 (4)	C10—H10B	0.9700
C1—C6	1.378 (3)	C10—C11	1.492 (4)
C2—C3	1.365 (4)	C11—H11A	0.9700
C3—C4	1.357 (4)	C11—H11B	0.9700
C4—H4	0.9300		
			
C6—N1—H1	112.6 (19)	N2—C7—N1	115.3 (2)
C7—N1—H1	120 (2)	N2—C8—H8A	111.2
C7—N1—C6	127.6 (2)	N2—C8—H8B	111.2
C7—N2—C8	127.1 (2)	N2—C8—C9	102.9 (2)
C7—N2—C11	120.7 (2)	H8A—C8—H8B	109.1
C11—N2—C8	112.3 (2)	C9—C8—H8A	111.2
F1—C1—C2	118.7 (2)	C9—C8—H8B	111.2
F1—C1—C6	118.6 (2)	C8—C9—H9A	110.3
C2—C1—C6	122.7 (2)	C8—C9—H9B	110.3
F2—C2—C1	120.2 (2)	H9A—C9—H9B	108.6
F2—C2—C3	120.7 (2)	C10—C9—C8	106.9 (2)
C3—C2—C1	119.0 (2)	C10—C9—H9A	110.3
F3—C3—C2	118.2 (3)	C10—C9—H9B	110.3
C4—C3—F3	121.0 (3)	C9—C10—H10A	110.4
C4—C3—C2	120.8 (3)	C9—C10—H10B	110.4
C3—C4—H4	120.1	C9—C10—C11	106.7 (3)
C3—C4—C5	119.7 (3)	H10A—C10—H10B	108.6
C5—C4—H4	120.1	C11—C10—H10A	110.4
C4—C5—H5	119.5	C11—C10—H10B	110.4
C6—C5—C4	120.9 (3)	N2—C11—C10	103.7 (2)
C6—C5—H5	119.5	N2—C11—H11A	111.0
C1—C6—N1	117.5 (2)	N2—C11—H11B	111.0
C1—C6—C5	116.8 (2)	C10—C11—H11A	111.0
C5—C6—N1	125.7 (2)	C10—C11—H11B	111.0
O1—C7—N1	122.3 (2)	H11A—C11—H11B	109.0
O1—C7—N2	122.5 (2)		

### Hydrogen-bond geometry (Å, °)

**Table:**

*D*—H···*A*	*D*—H	H···*A*	*D*···*A*	*D*—H···*A*
N1—H1···F1	0.77 (3)	2.27 (3)	2.672 (3)	113 (2)
